# Supplementary material for: Assessing Consumer Preferences for New Red-Pulp Kiwifruit: Application of a Choice Experiment between Different Countries
Source: Foods. 2023 Jul 27;12(15):2865. doi: 10.3390/foods12152865 (PMC10418330; doi:10.3390/foods12152865)
Supplement: Supplementary file 1 [file foods-12-02865-s001.zip › foods-2530450-supplementary.pdf]

Table S1. Italian sample distribution by REGION.

	Number	Percentage (%)
Abruzzo	7	2.3
Basilicata	3	1.0
Calabria	10	3.3
Campania	28	9.3
Emilia-Romagna	22	7.3
Friuli-Venezia Giulia	6	2.0
Lazio	27	9.0
Liguria	8	2.7
Lombardia	49	16.3
Marche	8	2.7
Molise	2	0.7
Piemonte	23	7.7
Provincia Autonoma di Bolzano Bozen - Trento	5	1.7
Puglia	20	6.7
Sardegna	8	2.7
Sicilia	24	8
Toscana	20	6.7
Umbria	5	1.7
Valle d'Aosta/Vallée d'Aoste	1	0.3
Veneto	24	8
**Total**	**300**	**100**

Table S2. Sample distribution by GENDER.

	Italy	Spain	French	Germany	Total
Female	52	51.2	52.2	52.7	52
Male	48.0	48.5	47.5	47.3	47.8
Other	0	0.3	0.3	0.0	0.2
**Total**	**100**	**100**	**100**	**100**	**100**

Table S3. Sample breakdown by AGE CLASS.

	Italy	Spain	French	Germany	Total
18-24	8.7	8.6	11.3	8.3	9.2
25-34	15.3	15.6	16.3	14.3	15.4
35-44	19.3	20.3	18.3	17.0	18.7
45-54	18.0	18.6	17.3	18.7	18.1
55+	38.7	36.9	36.9	41.7	38.5
**Total**	**100**	**100**	**100**	**100**	**100**

Table S4. Sample breakdown by LEVEL OF EDUCATION.

	Italy	Spain	French	Germany	Total
Elementary school, junior high school	33.0	15.9	11.6	10	17.6
High school, vocational school	47.7	38.9	52.8	54.3	48.4
University or higher qualification (e.g., doctorate)	19.3	45.2	35.5	35.7	33.9
**Total**	**100**	**100**	**100**	**100**	**100**

Table S5. Sample distribution by MONTHLY INCOME CLASS of the household.

	Italy	Spain	French	Germany	Total
Up to 1000 EUR	15.3	7	26.9	23	18.1
Up to 1500 EUR	26	18.3	19.9	15.7	20
Up to 2000 EUR	22	23.9	15.6	20	20.4
Up to 2500 EUR	12	20.6	13	13	14.6
Up to 3000 EUR	13	7.6	8.3	8.7	9.4
over 3000 EUR	11.7	22.6	16.3	19.7	17.6
**Total**	**100**	**100**	**100**	**100**	**100**

Table S6. Sample allotment by EMPLOYMENT.

	Italy	Spain	French	Germany	Total
Trader/merchant	0.7	1.7	2.3	6.3	2.7
Entrepreneur	0.7	3	0.7	2.3	1.7
Executive	2.7	2.3	2	3.7	2.7
Officer	2,3	4	11	2	4.8
Employee/teacher	18.3	20.9	23.3	25.7	22
Worker/clerk/farmer	18	3	6.3	5.7	8.2
Freelance	4.3	18.3	1.7	5.3	7.4
Other self-employed	2.3	1.3	2	2.0	1.9
Artisan	0.7	1.3	1.7	5.3	2.2
Houseworker	11.0	4.3	7.3	7.3	7.5
Student	6.3	5.6	6.3	3	5.3
Retired	17.7	14.3	23.3	24	19.8
Unemployed/looking for a job	13	15.3	7	3.7	9.7
Other	2	4.7	5.3	3.7	3.9
**Total**	**100**	**100**	**100**	**100**	**100**
